# Squalene-Based Influenza Vaccine Adjuvants and Their Impact on the Hemagglutinin-Specific B Cell Response

**DOI:** 10.3390/pathogens10030355

**Published:** 2021-03-17

**Authors:** Phuong Nguyen-Contant, Mark Y. Sangster, David J. Topham

**Affiliations:** David H. Smith Center for Vaccine Biology and Immunology, Department of Microbiology and Immunology, University of Rochester Medical Center, Rochester, NY 14642, USA; david_topham@urmc.rochester.edu

**Keywords:** squalene-based adjuvant, influenza A virus, memory B cells, hemagglutinin, antibody, germinal center

## Abstract

Influenza infections continue to cause significant annual morbidity and mortality despite ongoing influenza vaccine research. Adjuvants are administered in conjunction with influenza vaccines to enhance the immune response and strengthen protection against disease. Squalene-based emulsion adjuvants including MF59, AS03, and AF03, are registered for administration with influenza vaccines and are widely used in many countries. Squalene-based emulsion adjuvants induce a strong innate immune response, enhancing antigen presentation both quantitively and qualitatively to generate strong B cell responses and antibody production. They also diversify the reactivity profiles and strengthen the affinities of antibodies against the influenza hemagglutinin, increasing protection across virus clades. In this review, we consider the mechanisms of the enhancement of innate and adaptive immune responses by squalene-based emulsionSE adjuvants and the resulting increase in magnitude and breadth of hemagglutinin-specific B cell responses. We relate observed effects of SE adjuvants and current mechanistic understandings to events in responding lymph nodes. These insights will guide the rational design and optimization of influenza vaccines to provide broad and effective protection.

## 1. Introduction

Seasonal and pandemic influenza infections in humans continue to be a significant health threat, causing tremendous economic and societal burdens annually in the United States [1,2]. To date, the most efficient prophylactic approach to alleviate the morbidities and mortalities caused by influenza infections is immunization [3]. The antibody responses generated by vaccines provide short-term protection against reinfection. The induced memory B cells (MBCs) confer long-term protection, even after the antibody levels wane, by being recalled quickly upon re-exposure to pathogens [4,5]. Despite extensive research efforts, influenza vaccine development has been challenged by the intrinsic low immunogenicity of many influenza vaccines, particularly those formulated with subunit inactivated viruses or recombinant proteins. Reported vaccine effectiveness is often in the range of 40–60% and could be as low as 10–20% during the seasons when significant antigenic drift or antigenic shift occurs [6,7,8]. Therefore, adjuvants have been developed and co-administered with vaccines to augment the immune responses in target populations. Among the different influenza vaccines approved or in development for human use including live-attenuated virus, inactivated (whole-virus, ‘detergent’-split or subunit) vaccines, nucleic acid, and recombinant proteins, adjuvants most often benefit subunit inactivated virus- and recombinant protein-based vaccines. Due to safety concerns, however, influenza vaccine adjuvants currently have limited uses: (i) to augment the immune responses in subjects with reduced responsiveness such as infants and the elderly, and (ii) to increase seroconversion rates and to accelerate responses to vaccines that target novel influenza viruses with pandemic potential [9,10].

The primary goal of influenza vaccination is to induce the generation of neutralizing antibodies mainly targeting the surface glycoprotein hemagglutinin (HA) of the virus. HA proteins are structurally composed of two domains: the immunodominant head domain and the immunosubdominant stalk domain. The head domain has highly variable sequences, while the stalk is highly conserved across subtypes. The head and stalk domains account for the majority of antibody-binding epitopes on the two subunits HA1 and HA2, respectively, which are derived from cellular enzymatic cleavage of the precursor HA0 protein. Traditionally, the level of protection conferred by vaccines—the prerequisite for vaccine licensure—is assessed by the increase in circulating neutralizing antibodies measured by the hemagglutination inhibition and microneutralization assays at four weeks post injection. However, it has been recently recognized that the assessment of vaccine efficacy using these gold-standard assays is skewed toward short-term antibody response and largely underappreciates the role of vaccine-induced long-lasting MBCs. This biased assessment is particularly relevant since recent works have shown that adjuvants modulate both the magnitude and quality of MBC responses to provide better long-term protection.

Despite the century-long history of adjuvant development, few adjuvants have been approved for human use in the United States, mainly due to safety concerns. Among these, squalene-based emulsion (SE) adjuvants account for three out of less than ten adjuvants currently administered in licensed influenza vaccines: MF59, AS03, and AF03 [10,11]. In addition to their established safety profile and high tolerance in both adults and children, the licensing of SE-adjuvanted vaccines was supported by their increased vaccine immunogenicity, dose sparing, and broadened cross-strain neutralization compared to aluminum salts (Alum) [12,13,14,15,16,17,18,19,20,21]. While the search for new adjuvants, especially those based on rational design targeting pattern recognition receptors [22], is progressing at unprecedented pace, SE adjuvants are expected to remain in use in a foreseeable future because of their safety profile and adjuvanticities. With hundreds of millions of doses administered commercially worldwide, the widespread use of SE-adjuvanted vaccines underscores the importance of understanding their mechanisms of action. However, SE adjuvants are among the classical adjuvants developed with empirical approaches, together with complete Freund’s adjuvant (CFA), Alum, and saponin-based adjuvants (ISCOMs/ISCOMATRIX). As such, mechanisms of SE adjuvants only became known long after they were approved for human use, mainly through animal studies.

There have been several in-depth reviews on the mechanisms of action of MF59 adjuvant—the prototype of this adjuvant group—few on AS03, and none on AF03 [23,24,25,26,27]. However, the main body of the current literature focuses on how SE adjuvants modulate the innate immunity and the T cell component of adaptive immunity, leading to enhanced antibody response outcomes. There remains a gap in our understanding regarding how SE adjuvants modulate B cell activation, affinity maturation, and MBC generation—the critical processes that determine the quality of post-vaccination B cell immunity. There is also inadequate discussion of the differences in adjuvant-mediated B cell responses between primed subjects with prior exposure to influenza viruses versus naïve subjects. In this work, we aim to clarify the effect of SEs on antibody and MBC outcomes by providing a comprehensive review of their possible mechanisms during both the innate and adaptive immune responses. We propose models of how SE-modulated competition among preexisting MBCs or between MBCs and naïve B cells dictates the outcome of different vaccine regimes in naïve versus primed populations. Collectively, our discussions on SE mechanisms have implications on the future rational design of seasonal and pandemic influenza vaccines.

## 2. Squalene-Based Emulsion (SE) Adjuvant Overview

SE adjuvants are oil-in-water (o/w) emulsions formulated with completely metabolizable squalene droplets and nontoxic surfactants in the presence or absence of the immunocostimulatory α-tocopherol (Table 1) [12,28,29,30]. Squalene is present naturally in the human body as a direct precursor to cholesterol and is commercially derived from shark liver. The oil-in-water formulation with low nontoxic oil content, as opposed to the water-in-oil CFA emulsion, facilitates higher tolerance of SE adjuvants than CFA in humans.

SE adjuvants belong to the first of the three groups of adjuvants based on their dominant adjuvanticity mechanisms: delivery system that concentrates and/or targets the antigen to the antigen-presenting cells (APCs) (e.g., emulsions, Alum, nanoparticles); immunostimulatory molecule that directly engages and activates the innate cells (e.g., pattern recognition receptors-targeting adjuvants); or a combination of both [5]. Notably, adjuvants classified as a delivery system could also activate the innate immunity via inflammasome-dependent or -independent mechanisms. As such, o/w squalene adjuvants enable more effective antigen delivery and presentation to prime the immune system for a robust antibody and MBC response. Unlike CFA forming depots at the injection site that allow for gradual antigen release [31,32], SE adjuvants mainly act via depot-independent mechanisms [26]. Small oil droplet size enables efficient non-degradative endocytosis—a mechanism preferable to preserve intact antigen—and rapid transportation of antigens to the draining lymph nodes (LNs). Recent work by Pedersen et al. [33] demonstrated that SE-mediated rapid antigen delivery to the draining LNs mimics the kinetics of ‘bolus vaccination’.

## 3. SE Adjuvants Induce an ‘Immunocompetent Environment’ Leading to Enhanced Antigen Uptake and Delivery

Adjuvanted vaccines induce immunity by directly stimulating the innate immune system, which then generates signals required to activate the adaptive immunity. Since the magnitude and quality of local innate activation dictate the outcome of adaptive B cell responses in the draining LNs [34], we first summarize the current understanding of the action of SE adjuvants during the early innate immune responses.

**Figure 1:**
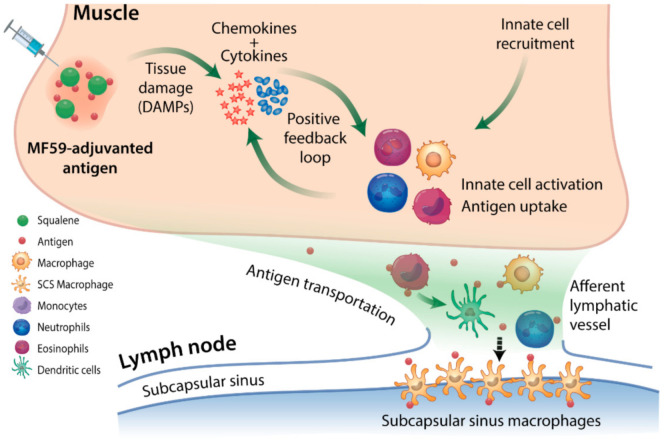
Immunocompetent environment induced by squalene-based emulsion (SE) adjuvants at the injection site. SE adjuvants cause local muscle cell damage (e.g., cell lysis by detergent-like molecules in the formulation), inducing the transient release of damage-associated molecular pattern (DAMP) signals such as ATP and double-stranded DNA, and the change in expression of as many as 891 genes in mice, compared to 312 genes by Alum at the injection site [35,42,43,44]. These DAMP signals bind to receptors on antigen-presenting cells (APCs) cells, trigger cytokine and chemokine secretion cascades as early as 3–6 h post-injection in mice [45]. This process leads to chemokine-driven infiltration of innate cells to the site of vaccine administration, generally peaking in 24 h, followed by a rapid decline by 72 h [38,45]. The signaling pathways triggered by SE adjuvants that drive the activation, APC differentiation, and lymph node migration of resident and recruited leukocytes remain unclear. Evidence suggests that these processes are inflammasome-independent and involve MyD88 and apoptosis-associated speck-like protein containing a caspase recruitment domain [46]. Activated innate cells secrete additional inflammatory molecules [32,33], continuing as a positive feedback loop at the injected muscle to generate a robust innate immune response. This mechanism allows SE adjuvants to create a localized transient ‘immunocompetent environment’ at the injection site, potently enhancing the antigen uptake and the activation of the APCs to migrate to the local lymph nodes and efficiently prime the adaptive system for B cell response [42]. The induction of an immunocompetent environment by SE adjuvants, however, is independent of immunogens [36]. The o/w emulsion vehicle itself appears to suffice [36].

Upon intramuscular injection, SE adjuvants induce a localized ‘immunocompetent environment’ mediated by damage-associated molecular pattern (DAMP) signals [35] (detailed in Figure 1) and characterized by cascades of chemokines and cytokines secreted by resident and recruited innate cells. This results in rapid and massive recruitment, followed by activation of different innate cell types including neutrophils, monocytes, eosinophils, dendritic cells (DCs), and F4/80+ macrophages [33,36,37], leading to enhanced antigen uptake.

Upon uptake by innate cells, antigens are transported to the draining LNs to initiate adaptive T cell and B cell responses [38]. Antigen delivery is achieved by active cellular carriers or via passive lymphatic drainage. The efficiency of antigen relocalization depends on the clearance kinetics of labeled adjuvants and immunogens at the injection site. The half-life of labeled SE adjuvants at the mouse quadriceps muscle varies from 1.5 h (for AS03) to 12.9 h (for MF59) [39] depending on the droplet size and composition of the emulsions. Antigen-positive innate cells subsequently peak in the proximal LNs at 24 h post-injection, which declines at 48 h to a baseline after 7–8 days in mice injected with MF59 SC [38]. Notably, although a substantial fraction of adjuvant is redistributed to the fat tissues and traces of adjuvants (less than 1%) could be detected systematically in spleen, spinal cord, brain, and blood [39], immunogen delivery was restricted to the draining LNs as shown in both mouse and rhesus macaques [38,40].

It is noteworthy that stable binding of antigens to the oil droplet vesicles with MF59 is thought not to be required for immune activation and efficient antigen relocation to the proximal LNs, although this is still unknown for AS03 [17,37,39]. Using the sucrose gradient centrifugation method, Ott et al. found no detectable binding of HSV antigens to MF59 emulsion in the oily phase after removing the aqueous phase [17]. Instead, the localized immunocompetent environment induced at the injection site described in Figure 1 is probably the primary mechanism of SE adjuvants. This theory is supported by the experiments in which antigens could be separately administered up to 24 h after the SE adjuvants, but not in the reverse order, without a deleterious effect on the elicited antibody response [17,37]. Notably, when the antigen is co-administered with MF59, most antigen-positive cells in the mouse draining LNs were loaded with labeled squalene 24 h post-vaccination [38]. Whether squalene-bound antigens are more prone to endocytosis by APCs or this double-positive population stems from separate uptakes of lymph-borne antigens and adjuvants by a limited number of APCs in the draining LNs awaits further study. If the former theory holds valid, stabilizing the binding of immunogens to squalene droplets may further improve vaccine immunogenicity. Such a strategy has recently been demonstrated for Alum and TLR agonist adjuvants [21,41].

O/w squalene emulsions appear to rapidly employ various recruited innate cells as carriers to facilitate efficient antigen transportation. Particularly, antigen-positive monocytes, DCs, and macrophages have been found in the proximal LNs as early as 3 h post-injection, with monocyte-to-DC differentiation playing an important role in MF59-mediated enhanced antigen presentation [47]. In addition to monocytes, other cell types such as neutrophils have also been proposed to contribute to antigen transportation and presentation. Strong immobilization of antigen-loaded neutrophils in local LNs was reported to be a unique effect of MF59 [33,38,40]. Neutrophils are also recruited, however, with lower frequency, to the injection sites of other adjuvants such as Alum, but not to their proximal LNs [33,40]. Furthermore, neutrophils are also capable of directly presenting antigens to T cells or activating memory CD4+ T cells under appropriate inflammatory conditions in vitro and in vivo [38,48,49,50]. However, neutrophil depletion does not abrogate the antibody response in MF59-immunized mice, suggesting that SE-induced antigen transportation is likely the overlapping function of different innate cell types [40].

In summary, SE adjuvants induce a localized inflammatory environment at the injection site, enabling rapid infiltration and maturation of various innate cells. Strong inflammatory molecule secretion by local and recruited innate cells provide positive feedback signals. This mechanism allows efficient SE-induced relocalization of immunogens in a ‘bolus’ fashion, as opposed to the gradual antigen release mediated by depot-dependent Alum or CFA. The following section will explore how the SE-formulated immunogen delivery with their inflammatory signature leads to enhanced B cell responses to influenza vaccines in both magnitude and quality.

## 4. SE Adjuvants Strongly Promote Adaptive B Cell Responses

**Figure 2:**
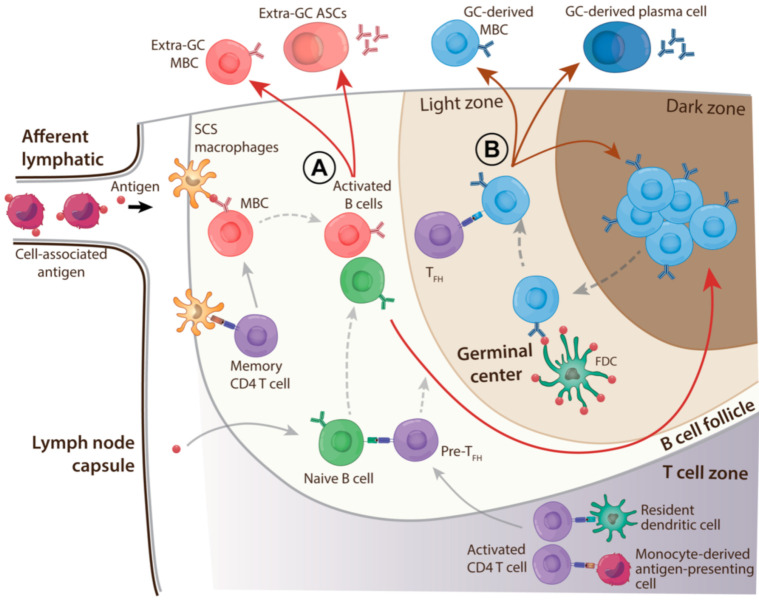
Overview of the HA-specific B cell response in a draining lymph node after administration of vaccine with squalene-based emulsion (SE) adjuvant. SE adjuvants promote antigen transport to lymph nodes by monocyte-derived cells. These cells, which cross sinus linings to enter lymph node parenchyma, have the potential to deliver intact antigen and also to serve as highly efficient antigen-presenting cells for CD4 T cell activation. Free lymph-borne antigen enters lymph node parenchyma via multiple routes such as trapping by subcapsular sinus macrophages and transfer for memory CD4 T cell activation or recognition by memory B cells (MBCs) and naïve B cells. Direct antigen transfer across lymphatic endothelial cells into lymph nodes also occurs. Abundant antigen availability in draining lymph nodes after administration with SE adjuvants diminishes B cell competition (for example, between MBCs and naïve B cells) for cognate antigen required for activation. MBCs and naïve B cells that “see” cognate antigen receive T cell help to drive activation and proliferation. Pathway options for activated B cells (A, red arrows) include (i) formation of extrafollicular antibody-secreting cells (ASCs); (ii) formation of extrafollicular MBCs; and (iii) entry into germinal center (GC) reactions. After maturation of immunoglobulin V region genes in GCs, GC B cells “test” their receptor affinity by competing for antigen held by follicular dendritic cells (FDCs). Successful acquisition of antigen from FDCs enables GC B cells to be stimulated by T cells and to repeat the maturation/selection cycle or differentiate into MBCs or antibody-secreting plasma cells and exit the GC (B, dark red arrows). The abundant free antigen in the lymph node is shuttled to FDCs, reducing the stringency of GC B cell selection after somatic mutation. In addition, strong CD4 T cell activation and formation of T follicular helper (T_FH_) cells promoted by SE adjuvants increases the availability of help for GC B cells and facilitates selection. Overall, SE adjuvants enhance the activation and affinity maturation of naïve B cells.

The kinetics and specificity of antibody and MBC responses in draining lymphoid tissues after inactivated influenza vaccination (IIV) reflect a complex process of naïve and/or MBC activation and differentiation. Briefly, HA-specific naïve and MBCs that have taken up antigens and been co-stimulated by activated T cells continue development via one of three pathways: (i) extrafollicular (germinal center (GC)-independent) antibody-secreting cell/plasmablast formation; (ii) extrafollicular MBC formation; or (iii) GC entry for affinity maturation (see Figure 2 and [51,52] for recent detailed reviews). Within GCs, B cells acquire mutations in immunoglobulin V genes during proliferation in the dark zone and then test the modified immunoglobulins in competition for antigen held by folicular dendritic cells (FDCs) in the light zone. B cells that successfully acquire antigen receive the CD4 T follicular helper (T_FH_) stimulation necessary to exit the GC as MBCs or long-lived plasma cells, or to repeat the dark zone/light zone cycle. GC processes are responsible for affinity maturation of B cell-expressed immunoglobulins, a critical requirement for protective B cell immunity.

### 4.1. B Cell Competition

HA-specific B cell responses to influenza vaccination in adults almost invariably involves activation of IgG-expressing MBCs formed over many years by multiple exposures to influenza virus through infection and vaccination. The presence of high affinity, vaccine HA-reactive MBCs in responding LNs adds an element of competition for vaccine antigen, which is a key determinant of the breadth of epitope recognition in the HA-specific response [53,54,55]. This competition can occur between (i) MBCs reactive to different epitopes on the variable HA head; (ii) MBCs reactive to the immunodominant HA head and those reactive to the conserved subimmunodominant stalk; and (iii) HA-reactive MBCs and the much less frequent naïve B cells reactive to novel HA epitopes. The above are examples of intramolecular competition; intermolecular competition can also occur, for example, between HA- and NA-reactive B cells when the molecules are linked as on viral envelope fragments or intact virions. HA-specific B cells that bind the HA component endocytose the complex of proteins and reduce the availability of NA for the activation of NA-specific B cells.

An important example of the impact of MBC competition on HA-specific antibody production is provided by a comparison of responses to unadjuvanted seasonal and pre-pandemic influenza vaccines and vaccines that target the novel HAs of avian influenza viruses that have caused human infection and are considered to have pandemic potential (referred to as pre-pandemic influenza vaccines). The response to the seasonal influenza vaccine is typically mediated exclusively by the large numbers of pre-existing HA head-reactive MBCs. Stalk-reactive MBCs are outcompeted for antigen, reducing activation and limiting production of anti-stalk antibodies [56]. In contrast, the head domains of pre-pandemic influenza virus HAs (e.g., H5, H7) are novel to most adults and reactive MBCs are not present. As a result, unadjuvanted pre-pandemic influenza vaccines activate pre-existing MBCs reactive to the stalk and there is early anti-stalk antibody production [57,58,59]. This is why sequential administration of chimeric HAs with exotic head domains is effective as a universal influenza vaccination strategy [60].

### 4.2. Mechanisms of Enhancement of B Cell Responses by SE Adjuvants

Antigen availability to B cells in responding LNs is an important determinant of the magnitude and character of B cell responses. Multiple studies have demonstrated that sustained antigen delivery to LNs, for example, by use of controlled release devices, enhances the magnitude and duration of GC reactions regardless of antigen depot formation [61,62,63]. The depot-forming adjuvant alum gradually releases antigen, but antigen responses are lower and have narrower specificities than with the depot-independent MF59 and AS03 adjuvants, indicating that antigen retention at the injection site and prolonged release alone do not account for SE adjuvant potentiation of B cell responses [31].

Many studies of adjuvant mechanisms underscore the significance of the immunostimulatory environment induced by adjuvant at the injection site and in draining LNs [37,42]. Optimal vaccine effectiveness is likely to reflect the synergistic effects of an inflammatory environment and prolonged delivery of a sufficient antigen load. As we have described above, establishment of a strong inflammatory state by SE adjuvants at the site of administration induces a robust influx of antigen-transporting cells that mediate highly efficient transport of endocytosed antigens to draining LNs. Under weaker inflammatory conditions and with fewer antigen-transporting cells, a higher proportion of antigens are likely to travel to LNs as immune complexes or free molecules. The form of antigen as it is transported to draining LNs can influence the amount of antigen lost to degradation, the speed of antigen delivery to responding cells, and the adaptive immune pathways activated by antigen [64,65]. In the following sections, we consider the reported effects of SE adjuvants in enhancing the magnitude, breadth of reactivity, and affinity maturation of HA-specific B cell responses induced by vaccination. We relate these effects to modulation of specific events in responding LNs by the two general mechanisms of response enhancement by SE adjuvants, namely, induction of an inflammatory state and the associated increase in the availability of antigen for immune response activation.

The HAs of pre-pandemic avian influenza viruses were initially thought to be poorly immunogenic [66]. This was based on the observation that priming naïve subjects with unadjuvanted pre-pandemic influenza vaccine and boosting with the same vaccine after 3–4 weeks generally did not produce protective antibody levels (HAI titers of ≥1:40). In other words, the level and/or affinity of anti-HA head antibodies after the prime-boost regimen remained insufficient for effective virus neutralization. Increasing the HA dose of adjuvanted H5N1 and H7N9 vaccines only modestly increased neutralizing antibody titers after the 2-dose regimen [67,68,69]. A surprising observation was that priming and boosting naïve subjects with unadjuvanted H5 or H7 generated strong subtype-neutralizing antibody responses when the time until boosting was extended to six months or more and even to several years [70,71]. The long-interval boost apparently allowed an on-going immune response to the priming HA vaccine to generate enough high affinity, head-reactive MBCs to outcompete stalk-reactive MBCs and dominate the response to the boost. Recent studies have comprehensively characterized on-going GC reactions after avian HA priming to generate increasingly affinity-matured HA head-reactive MBCs [72]. It is now established that an interval of 8–12 weeks between priming and boosting with an unadjuvanted pre-pandemic influenza vaccine is necessary to obtain an optimally protective antibody response to the boost. Although a single priming dose of pre-pandemic vaccine given with SE adjuvant generates little protective antibody, it does prime for strong protective antibody production when a booster dose (with or without adjuvant) is given after as little as three weeks [67,68,69]. Clearly, SE adjuvant modulates the B cell response to pre-pandemic HA priming to accelerate the process of head-reactive MBC production.

Khurana and colleagues [73,74] used the genome fragment phage display library technique to provide additional evidence that SE adjuvants promoted B cell responses to novel HA head epitopes. The antibody response to H5 epitopes was followed after two doses of an H5N1 vaccine given with SE or Alum adjuvants or unadjuvanted. SE adjuvants strongly enhanced epitope spreading and a shift in antibody binding to predominantly target HA1 (head) epitopes over HA2 (stalk) epitopes. In addition, antibody affinity for the H5 was increased 10–20-fold by vaccines with SE adjuvants compared with 5-fold for unadjuvanted vaccines, consistent with strong GC stimulation. Notably, the increase in H5 affinity with the AS03 adjuvant was selective for the novel HA1 and unchanged for the more conserved HA2 [73,74]. Support for increased naïve B cell activation by SE adjuvants was provided by Galson and colleagues [75], who sequenced BCRs of circulating antibody-secreting plasmablasts collected seven days after a single dose of AS03-adjuvanted seasonal influenza virus vaccine, two doses of an AS03-adjuvanted 2009 pandemic H1N1 influenza virus vaccine, or unadjuvanted forms of the vaccines. Relatively unmutated plasmablast BCR sequences, which suggests plasmablast formation from naïve B cells, were most frequent after the AS03-adjuvanted vaccines.

As we have described above, SE adjuvant effects stem from events at the site of injection that result in rapid and increased delivery of antigen, primarily cell-associated, to drive B cell responses in draining LNs. A major initial effect of SE adjuvants on LN responses is enhanced CD4 T cell activation, which is the basis for (i) potent T_FH_ cell differentiation; (ii) increased naïve B cell activation; and (iii) strong GC activity (see Figure 2 for overview) [33,38,76]. The large numbers of APCs that migrate to LNs from sites of administration of antigen with SE adjuvants would substantially augment CD4 T cell activation by LN resident DCs [77]. In addition, the migrating antigen-bearing cells might also deliver intact antigen that could greatly supplement the amount of antigen that is typically available in LNs for immune response activation. This form of antigen delivery to LNs is not well understood, but antigen could potentially be made available at locations that facilitate uptake by naïve or MBCs or by DCs that activate CD4 T cells [65,78,79]. Preferential selection of the T_FH_ differentiation pathway by activated CD4 T cells might be promoted by the early production of IL-6 in LNs that is associated with SE adjuvants [37,80]. Cell-borne intact antigen might be especially important for generating responses to novel glycosylated HA proteins that cannot readily form immune complexes with pre-existing antibodies. This situation would limit antigen uptake from afferent lymph and its display for B cell recognition as well as increase the likelihood of antigen loss by degradation [64].

When antigen is limited in a responding LN, B cells must compete for the antigen that is essential for their activation. Often, the activation of naïve B cells reactive to a particular HA epitope is limited or prevented by the presence of expanded populations of MBCs that bind with high affinity to different epitopes on the same HA molecule. An example of this is the original antigenic sin response to influenza virus infection, where the initial antibody response to the HA is primarily mediated by preferential activation of pre-existing MBCs specific for HA epitopes “seen” on previous infecting viruses. This is frequently sufficient to clear the infecting virus and terminate the response before naïve B cells specific for novel HA epitopes are activated [54,81]. Rapid delivery of abundant antigen to a responding LN, as when antigen is administered with SE adjuvants, would be expected to diminish B cell competition and promote naïve B cell activation. Notably, resting MBCs preferentially accumulate in the outer LN cortex where they are well-placed to receive antigen captured from afferent lymph by subcapsular sinus macrophages [82]. It is not known whether some of the antigen administered with SE adjuvants enters LNs via pathways that favor recognition by naïve B cells. Enhanced CD4 T cell activation by SE adjuvants would increase the likelihood of strong “second signal” interactions with naïve B cells that drive B cell activation and the differentiation of pre-T_FH_ cells into mature T_FH_ cells [80].

GC reactions support affinity maturation of the B cell response and generate MBCs and antibody-secreting cells. In mouse studies, GCs formed more rapidly when antigen was administered with SE adjuvants, reflecting rapid antigen transfer to LNs and seeding of GC reactions in B cell follicles by activated B cells and T_FH_ cells [33]. Importantly, abundant antigen in the LN also facilitates antigen delivery to FDCs, which display the antigen for B cell selection in GCs [78]. After somatic mutation of their BCRs, B cells must take up and process antigen held by FDCs in order to receive survival signals from T_FH_ cells. Abundant antigen on FDCs would increase the likelihood that activated naïve B cells specific for novel epitopes (potentially with relatively low affinity BCRs) compete successfully for antigen and continue the process of affinity maturation. This scenario, in combination with increased activation of naïve B cells, could account for accelerated production of affinity-matured HA head-reactive MBCs when pre-pandemic influenza vaccines are given with SE adjuvants in humans.

## 5. Conclusions

Numerous human, non-human primate, and mouse studies have provided considerable information on the benefits of SE adjuvants in enhancing B cell responses to vaccination. This work has also established a broad understanding of mechanisms underlying SE adjuvant effects, but many details remain unclear. Our goal in this review was to focus on SE adjuvants in the context of influenza vaccination and, in particular, their effect on the HA-specific B cell response. We related, as far as possible, the effects of SE adjuvants on circulating antibody levels after vaccination and current mechanistic insights to events in responding lymph nodes. Key SE adjuvant effects in lymph nodes, reflecting early events at the site of antigen administration, are (i) enhanced CD4 T cell/T_FH_ cell formation; (ii) increased naïve B cell activation; and (iii) rapid and strong GC activity that facilitates ongoing positive selection and affinity maturation of activated naïve B cells. As described above, a pre-pandemic H5 or H7 vaccine given with SE adjuvant (in contrast to nonadjuvanted vaccine) primes for a protective response to a boost after only three weeks. This clearly requires activation, by the priming dose, of naïve B cells specific for novel H5 or H7 head epitopes and progression of these cells through GC reactions to form MBCs. We previously suggested that bolus-type antigen delivery with adjuvant (as occurs with SE adjuvants) increases the magnitude of early GC activity and HA head-reactive MBC formation after pre-pandemic influenza vaccination [66]. Increased loading of antigen on FDCs in GCs, increasing the likelihood of positive selection of relatively low affinity head-reactive B cells, is likely to be important in this process.

Additional work is required to fully explore the potential of SE adjuvants to promote epitope spreading by increasing antigen availability in different locations in responding lymph nodes. Immunodominance hierarchies play an important role in determining the relative strengths of B cell responses to particular domains and antigenic sites on the HA molecule. For instance, antibody responses target epitopes on the immunodominant head domain much more strongly than those on the subdominant stalk domain; an immunodominance hierarchy also exists for antigenic sites on the HA head [83]. Response patterns are further complicated by the composition of the HA-reactive MBC pool established in each individual by a lifetime of exposure to influenza subtypes and variants through infection and vaccination [84]. B cell competition for antigen plays a role in every individual’s HA-specific antibody response. Influenza vaccines with SE adjuvants could be of great value in overcoming the negative effects of B cell competition such as those that inhibit responses to novel epitopes. Although it has been established that AS03 induces a stronger antibody response than MF59, perhaps attributed to the additive effect of the immunocostimulatory α-tocopherol, a knowledge gap exists regarding the immunopotentiation of AF03 relative to AS03 or MF59. It will also be important to compare responses to vaccines given with SE adjuvants and those generated by new vaccine technologies with potential adjuvant activity [85]).

## Figures and Tables

**Table 1:** Licensed vaccines co-administered with squalene-based emulsion (SE) adjuvants.

Adjuvants	Component	Vaccines	Trade Name	Use (Age Group)	Manufacturer
MF59	Squalene; polysorbate 80; sorbitan trioleate	Seasonal influenza vaccine	FLUAD FLUAD Quadrivalent	65 years and older	Novartis
		A/H1N1 pandemic influenza vaccine	Forcetria Celtura	6 months and older	Novartis
AS03	Squalene; α-tocopherol; polysorbate 80	A/H1N1 pandemic influenza vaccine	Pandemrix Prepandrix	6 months and older 18 years and older	GlaxoSmithKline (GSK)
AF03	Squalene; polyoxyethylene cetostearyl ether; mannitol; sorbitan oleate	A/H1N1 pandemic influenza vaccine	Humenza	6 months and older	Sanofi
